# Modelling Sporadic Alzheimer’s Disease Using Induced Pluripotent Stem Cells

**DOI:** 10.1007/s11064-018-2663-z

**Published:** 2018-11-01

**Authors:** Helen A. Rowland, Nigel M. Hooper, Katherine A. B. Kellett

**Affiliations:** Division of Neuroscience & Experimental Psychology, School of Biological Sciences, Faculty of Biology Medicine and Health, University of Manchester, Manchester, UK

**Keywords:** Sporadic Alzheimer’s disease, Induced pluripotent stem cells, Neurons, Glial cells, Genetic stratification, Environmental risk factors

## Abstract

Developing cellular models of sporadic Alzheimer’s disease (sAD) is challenging due to the unknown initiator of disease onset and the slow disease progression that takes many years to develop in vivo. The use of human induced pluripotent stem cells (iPSCs) has revolutionised the opportunities to model AD pathology, investigate disease mechanisms and screen potential drugs. The majority of this work has, however, used cells derived from patients with familial AD (fAD) where specific genetic mutations drive disease onset. While these provide excellent models to investigate the downstream pathways involved in neuronal toxicity and ultimately neuronal death that leads to AD, they provide little insight into the causes and mechanisms driving the development of sAD. In this review we compare the data obtained from fAD and sAD iPSC-derived cell lines, identify the inconsistencies that exist in sAD models and highlight the potential role of Aβ clearance mechanisms, a relatively under-investigated area in iPSC-derived models, in the study of AD. We discuss the development of more physiologically relevant models using co-culture and three-dimensional culture of iPSC-derived neurons with glial cells. Finally, we evaluate whether we can develop better, more consistent models for sAD research using genetic stratification of iPSCs and identification of genetic and environmental risk factors that could be used to initiate disease onset for modelling sAD. These considerations provide exciting opportunities to develop more relevant iPSC models of sAD which can help drive our understanding of disease mechanisms and identify new therapeutic targets.

## Introduction

The potential of using human neurons derived from induced pluripotent stem cells (iPSCs) for in vitro studies has revolutionised research of dementia and other neurodegenerative diseases as many of the challenges that are posed from studying complex disease pathways in the brain could potentially be unravelled in a cell-culture dish. The number of people with Alzheimer’s disease (AD), the most common form of dementia, is increasing rapidly within our ageing population. While progress has been made in the understanding of disease pathogenesis, we still do not have any disease modifying treatments. Access to human neurons derived from iPSCs provide a significant advantage for translational research but these models still need to be refined for us to be able to model the more complex facets of AD.

AD is characterised by amyloid plaques, composed of amyloid-β (Aβ), and neurofibrillary tangles (NFTs), composed of hyperphosphorylated and truncated tau, in the brain. The amyloid cascade hypothesis, proposed in 1992 [1], whereby the production of Aβ from the sequential β- and γ-secretase cleavage of the amyloid precursor protein (APP) drives disease progression resulting in the formation of NFTs and ultimately toxicity and neuronal cell death, remains the principal hypothesis for investigation [2]. Much of the published evidence supporting this hypothesis comes from studies investigating changes that occur due to increased Aβ production as a result of mutations in either APP or in presenilin 1 (*PSEN-1*) or *PSEN-2*, components of the γ-secretase complex, that occur in familial AD (fAD). In fAD these mutations drive disease progression due to increased Aβ load, but while these mutations have provided a useful research tool, particularly in the development of transgenic animal models, it is becoming more evident that the disease mechanisms driving sporadic AD (sAD) [3] which accounts for > 95% of all AD cases, may be due to more subtle alterations in multiple pathways.

The genetics of sAD have been widely investigated and a number of genes have been indicated as potential risk factors for disease development. The presence of the ε4 isoform of Apolipoprotein E (*APOE*) remains the most significant risk factor for developing sAD with those heterozygous for the ε4 allele having an approximately fourfold increased risk of developing AD, while those homozygous for ε4 have an approximately 12-fold increased risk [4]. More recently, triggering receptor expressed on myeloid cells 2 (*TREM2*) has been identified by whole-genome sequencing as another significant risk factor, with loss of function mutations in *TREM2* suggested to cause a 2–3 fold increased risk of developing sAD [5, 6]. While *APOE* and *TREM2* variants could be considered high-risk genes, several other low-risk genes have been identified by genome-wide association studies (GWAS) (extensively reviewed by Raghavan and Tosto [7]). The advent of GWAS studies was predicted to reveal the components of genetic risk in sAD and bring about a new understanding of the disease. However, while a number of genes were identified and validated in separate GWAS studies, their contribution to the overall development of disease pathology is still not fully understood. Interestingly, however, as a number of these genes, including *APOE* and *TREM2*, are involved in multiple key pathways linked to sAD, it suggests that understanding the genetics of AD may enable us to significantly progress our understanding of an individual’s risk of developing the disease.

With the advent of iPSC technology to produce human neurons there has been a number of studies using iPSC-derived neurons to investigate AD. Along with this has come a wealth of review articles to compare differentiation protocols, discuss disease modelling and review evidence from different research studies. The field is advancing rapidly but with the majority of these primary research studies describing models derived from patients with familial mutations, it still appears that we are a long way from really understanding the more complex sAD. In this review we will first discuss the existing studies using iPSC-derived stem cell models and compare those using cells derived from fAD patients to studies using cells derived from control individuals or sAD patients. Second, we will acknowledge the limitations of iPSC-derived models and discuss the progress that is needed to build better research models. Finally, we will address whether further genetic stratification and/or the introduction of environmental risk factors may enable us to realistically study sAD in iPSC-derived cellular models.

## Investigating sAD Using iPSC-Derived Neurons

To date, most AD models using iPSC-derived neurons have focused on familial forms of AD (fAD), the progress and outcomes of which have recently been well reviewed [8]. These studies, using functional, electrophysiologically active neurons, have predominantly used iPSCs from patients diagnosed with AD with either fAD mutations in *APP* or *PSEN1*, or from patients with Down’s syndrome where the duplication of the *APP* gene results in increased Aβ driving early-onset AD. All studies have, so far, shown an increase in Aβ; either in total Aβ or, more specifically in Aβ42 only, resulting in an increase of the Aβ42:40 ratio. An increase in the aggregation-prone Aβ42, and in the Aβ42:40 ratio, accelerates the disease through the production of toxic, oligomeric Aβ species and the formation of amyloid plaques. Other changes have also been observed in these studies including an increase in or altered processing and localisation of APP, an increase in tau and tau phosphorylation, and the activation of GSK3β, a physiological kinase of tau. In this section we review the current studies using iPSC-derived neurons from sAD patients and discuss their findings in terms of future modelling of sAD.

### Aβ Production in sAD

The proteolytic processing of APP has been shown to change over time in iPSC-derived neurons. In cortical neurons, β-secretase cleavage of APP was not apparent until deep-layer (TBR1-positive) neurons were present in culture, with the expression of the β-secretase (β-site APP cleaving enzyme-1; BACE1) also increasing. This is in contrast to the α-secretase, responsible for the non-amyloidogenic processing of APP, which was present in the neural progenitor stage of development and throughout neuronal maturation, although with a tendency to decrease after day 60 [9]. This highlights the importance of using cultures of appropriate maturity for investigating disease pathways. It should also be noted that selection of cellular subtypes are important in model selection as neurons directed to a rostral, cortical fate are more sensitive to Aβ than neurons directed to a caudal, hindbrain/spinal cord fate. This may not be surprising as the rostral, cortical neurons are known to be affected during AD whereas those of a caudal fate are relatively spared in the disease [10].

Limited studies have utilised iPSCs from patients with sAD. Initial studies looked to compare the levels of Aβ between neurons derived from sAD and fAD patient lines and compare these results to controls [3, 11]. The results of these, and later studies have demonstrated, in iPSC-derived neurons, increased Aβ levels [3, 11], altered Aβ42:40 ratios [12] and increased APP expression [12] in sAD patients compared to matched controls, consistent with that seen in fAD models. However, this work also revealed that these changes are not consistent in all sAD patients [3, 11]. As alluded to in the introduction, sAD is a complex disease with activation of a number of key disease pathways and a multitude of potential risk factors, both genetic and environmental, so it is not surprising that there is a lack of consistency between cell lines derived from different patients. While the genetics driving fAD cause early onset AD when patients are < 60 years old, the genetic risk factors identified in sAD patients lead to a later age of onset AD (late onset AD, LOAD). These genetic risk factors, which will be discussed in further detail in “Genetic Stratification for sAD” and “Environmental and Genetic Risk Factors” sections, do not drive disease progression in the same way as in fAD, and it is likely to be a combination of genetic and environmental risk factors that leads to the development of sAD. For the purpose of this review, we will discuss studies undertaken on iPSCs derived from sAD and LOAD patients under the term sAD; we have included further details of all studies, including details of the cell lines used, and their effects in Table 1 for clarification.

**Table 1:** iPSC models from patients with sporadic AD

sAD line	Cell type(s)	Phenotype(s)	Disease APOE genotype	Additional culture conditions	Environmental or genetic risk factor	Experimental results
Balez et al. [154]	Neurons	↑ Aβ42			H_2_O_2,_ NO	↑ neurite retraction, apoptosis, hyper-excitable Ca^2+^ signalling
Birnbaum et al. [29]	Neurons (iN)	↑ ROS, ↑ DNA damage	E3/E3, E3/E4			
Chen et al. [86]	Neurons			3D neuro-spheroid		Neuronal dysfunction similar to AD brain tissue
Duan et al. [123]	BFCNs	↑ Aβ42:40	E3/E4		Ionomycin, L-glutamate	↑ excitotoxicity
Hossini et al. [28]	Neurons	↑ GSK3β				
Israel et al. [11]	Neurons	↑ Aβ40, ↑ p-tau, ↑ GSK3β^a^	E3/E3	+ Human astrocytes (Lonza)		↑ very large early endosomes
Jones et al. [55]	Astrocytes	Altered S100β, EAAT1, GS and inflammatory mediators expression and localisation	E4/E4			
Kondo et al. [33]	Neurons, Astrocytes	↑ ER stress, ↑ OS, ↑ Aβ oligomers^a^		+ astrocytes of same iPSC line		↑ ROS ↑ Aβ oligomers
Lee et al. [85]	Neurons			3D neuro-spheroid		
Lin et al. [56]	Neurons Astrocyte Microglia-like	↓ Aβ uptake, ↑ Aβ42	E4/E4, E3/E3	Organoids		↑ p-tau
Ochalek et al. [12]	Neurons	↑ Aβ42:40, ↑ APP, ↑ GSK3β, ↑ p-tau			Aβ oligomers, H_2_O_2_	↑ sensitivity to OS
Young et al. [30]	Neurons				SORL1	
Balez et al. [154]	NSCs	↓SORL1	E4/E4			

*BFCNs* basal forebrain cholinergic neurons, *ER* endoplasmic reticulum, *NSC* neural stem cells, *OS* oxidative stress, *ROS* reactive oxygen species

^a^Phenotypes not observed in all sAD lines in study

### Aβ Degradation in sAD

While the majority of studies using iPSC-derived neurons have focussed on increased Aβ production, few, if any, have considered the impact of changes in Aβ clearance for modelling AD. Previous work has demonstrated, in a study of the CNS of cognitively normal compared to sAD patients, that there was no difference in the production of either Aβ40 or Aβ42. What was identified, however, was a significant impairment of Aβ clearance in those with AD [13]. The clearance of Aβ occurs in several different ways: the interstitial fluid (ISF) drainage pathway, microglial and macrophage phagocytosis, transcytosis across the blood–brain barrier (BBB), autophagy and proteolytic degradation. All of these pathways have been implicated in sAD and therefore warrant further investigation in iPSC-derived patient cells to determine how they may contribute in the development of sAD [14].

Neuronal Aβ degradation is primarily brought about through proteolytic degradation and autophagy. Several proteases have been recognised for their ability to degrade Aβ, a large number of which are metalloendopeptidases, including neprilysin (NEP) and insulin degrading enzyme (IDE) which appear to be the largest contributors [15]. Clearance and degradation of Aβ by autophagy is disrupted in AD causing increased intracellular Aβ which can be toxic to neurons [16, 17]. Neurons may, however, have a relatively small role to play in terms of Aβ clearance compared to surrounding glial cells. The contribution of enzymatic degradation by astrocytes may be a major contributing factor and there is evidence to suggest that APOE, the most significant genetic risk factor for sAD, affects proteolytic degradation of Aβ by astrocytes via NEP and IDE [18, 19]. Of the other pathways mentioned, Aβ clearance via the ISF drainage pathway may play a larger role than previously considered [20] and clearance through the BBB, mediated by Aβ chaperone proteins, is disrupted in AD [21]. Both of these pathways link to known genetic risk factors of LOAD and indicate additional avenues for investigation requiring modelling of the BBB as part of the neurovascular unit (NVU) which is the interface between the neural and vascular cells. The role of glia and other cell types in the brain and CNS and their potential role in the development of AD will be discussed further in “Building Better Models for sAD” section and the implications of genetic risk factors for sAD will be discussed in “Genetic Stratification for sAD” section.

### Tau Pathology in sAD

iPSC-derived neurons from fAD cell lines over-expressing APP (cell lines from patients with Down’s syndrome), or with *APP* mutations, have increased total tau and increased tau phosphorylation at the following key sites; Thr231, Thr181, Thr212, Thr205, Ser202 and Ser396 [12, 22–26]. These studies indicate a link between Aβ production and abnormal tau phosphorylation, which correlates with the amyloid cascade hypothesis. Tau phosphorylation, however, was unchanged in neurons derived from patients with a *PSEN1* mutation [24] and a recent study demonstrated that the increase in tau phosphorylation observed in neurons derived from a patient with Down’s syndrome was unchanged following correction of the *APP* gene dosage [27]. These latter studies therefore suggest that tau may have a role in the disease that is independent of that driven by Aβ pathology.

Limited studies have provided the measurement of tau and tau phosphorylation in iPSC-derived neurons from sAD cell lines. Initial studies demonstrated an increase in both total tau and phosphorylated tau compared to control cell lines; although these were limited in their measurement of the Thr231 phosphorylation site only [11, 28]. More recently, the analysis of a number of other key sites in iPSC-derived neurons from a sAD cell line has demonstrated an increase in tau phosphorylation at Thr205, Thr181, Thr403, Ser202, Ser400 and Ser404 compared to control [12]. Interestingly, this study also evaluated these sites in a *PSEN1* mutant cell line and demonstrated a similar increase in this fAD cell line [12], an observation that is in contrast to the lack of tau phosphorylation in the *PSEN1* fAD cell line described above [24]. In addition to the direct measurement of tau phosphorylation, the activation of glycogen synthase kinase (GSK) 3β, a tau-kinase that has been demonstrated to be responsible for abnormal tau phosphorylation in AD, was increased in iPSC-derived neurons from sAD cell lines in a manner similar to that observed in fAD [11, 12]. However, while the demonstration of increased tau phosphorylation in sAD is promising for the development of sAD models, not all studies have seen these changes, with no alterations in total or phosphorylated tau being observed between sAD and control despite observed alterations in Aβ [29]. As discussed in further detail in “Neuronal Maturation” section, the relevance of the observed changes in tau may be limited by the maturity of the neurons and more specifically developmental regulation of tau and the expression of the immature 3-repeat isoform of tau, rather than the mature 4-repeat isoform that is implicated in the pathogenesis of AD.

### Development of Other Phenotypes in sAD

Other AD-related phenotypes such as ER stress [3] and oxidative stress [3, 29] have also been identified in iPSC-derived neuronal models using sAD cell lines. A recent study demonstrated oxidative stress and altered mitochondrial protein expression in the absence of Aβ and tau pathology in sAD [29]. This study therefore highlights the possibility of alternative or additional mechanisms of disease pathogenesis, which need to be investigated to really understand the onset and progression of sAD. Identification of such disease mechanisms may be helped by the identification of changes in AD-associated genes, including genes identified by GWAS. Alterations in gene expression in iPSC-derived neurons from a sAD patient compared to control neurons identified up-regulation of AD-related pathways e.g. the response to oxidative stress, and alterations in proteasome complex units [28], indicating that genetic stratification of sAD patients may enable us to reduce the variation between cell lines, and promote a more consistent approach to selecting appropriate cell lines to model specific aspects of sAD. Indeed, stratification using variants of *SORL1*, a known genetic risk factor for AD, has demonstrated the potential of such an approach [30] and several studies have investigated the *APOE* genotype of their cell lines. Genetic stratification of iPSC lines and its potential to improve sAD cell models will be discussed further in “Genetic Stratification for sAD” section.

### Induction of AD Using Aβ Oligomers

As the amyloid cascade hypothesis points to Aβ as the trigger for the development of AD much previous work has sought to identify the toxic species of Aβ. Over recent years Aβ oligomers (AβOs), small aggregates of Aβ, have been identified as the toxic entity in initiating disease pathogenesis. Therefore, the addition of exogenous Aβ to initiate disease mechanisms in iPSC-derived neurons could be considered as a proof-of-principle experiment to validate this model for the study of AD. Here, we briefly discuss three studies using iPSC-derived models and the addition of AβOs. Kondo et al., [3] demonstrated that iPSC-derived neurons from a fAD patient with an *APP* mutation (E693Δ) and one sporadic AD line had increased intracellular AβO accumulation with associated ER and oxidative stress [3]. The effect of exogenously applied AβOs to iPSC-derived neurons was also investigated using either secreted oligomers from 7PA2 cells [31] or a synthetic oligomer preparation [32]. These studies demonstrated that addition of secreted AβOs to a control cell line induced AD-associated changes including an increase in tau phosphorylation [31] but in contrast, addition of synthetic AβOs to a control cell line had no effect [32]. Addition of synthetic AβOs did, however, result in an increase in toxicity in neurons derived from both sporadic and familial AD cell lines [32]. These studies, while highlighting the inconsistencies between different sAD cell lines, indicate that exogenous application of AβOs could be a useful mechanism for induction of AD-associated pathways in the study of sAD.

## Building Better Models for sAD

The development and refinement of iPSC-derived models for AD is ongoing and significant advances have been made. However, iPSC models and particularly iPSC-derived neurons have their limitations and using them to study sAD is particularly challenging. In this section, we highlight the major limitations of existing models and discuss how these may be overcome to generate better models for AD research, particularly for sAD.

### Neuronal Maturation

The loss of the ageing profile following reprogramming of fibroblasts to iPSCs is now well documented. Studies in iPSC-derived neurons indicate that these neurons are immature in phenotype [33] and lack markers of ageing [34]; an issue which has clear implications when using neurons derived from these cells to model sAD, where the primary risk factor is age. Defining ageing is in itself challenging, and among many cell types, neuronal ageing has been characterised by various markers of senescence, loss of nuclear lamina (providing mechanical stability and epigenetic regulation and linked with premature ageing, for example progerin in Hutchinson-Gilford Progeria syndrome), loss of heterochromatin markers (demonstrating DNA damage) [34, 35] in addition to other types of oxidative stress.

Whether this ‘resetting’ of iPSCs and the resulting immature neurons is an issue for AD research is, however, a source of debate. Robust differentiation protocols to generate different types of neurons from iPSCs now exist, and with an appropriate period of maturation, electrophysiologically active neurons can be produced [23]. More importantly, these cells can produce functional neural networks, a key requirement if we are to understand the pathogenesis of AD [36]. The ‘ageing’ of neurons and the use of age-related proteins to induce ageing are discussed in the “Ageing” section but other alternative approaches in the method of reprogramming have also been proposed. Direct reprogramming, or transdifferentiation, which omits the conversion to iPSCs and directly reprograms fibroblasts to neurons [37] produced neurons with an ageing signature [4, 38]. While maintaining an ageing signature in neurons is a distinct advantage, direct reprogramming approaches are less well characterised and less efficient. In addition, differentiation of iPSCs to cortical neurons has been shown to mimic development in vivo, including the spontaneous generation of glial cells such as astrocytes, and may therefore have some advantages in modelling AD progression [39]. Ghaffari et al. provides a detailed review and comparison of iPSC differentiation and direct reprogramming to generate models for neurological diseases including AD [40].

One of the key limitations of iPSC-derived ‘immature’ neurons to study AD, and other tauopathies, is the lack of a mature tau isoform profile in these neurons. Tau is alternatively spliced to produce six isoforms which differ by the expression of the number of N-terminal repeats (0N, 1N and 2N) and the number of microtubule binding regions (3R and 4R). This splicing is developmentally regulated, with foetal expression of the 0N3R tau and expression of the longer tau isoforms considered a mature (adult) tau profile. Disruptions in tau splicing and an increase in 4-repeat tau isoforms are seen in disease with 4-repeat tau being more prone to aggregation. iPSC-derived neurons predominantly express 3-repeat tau and, while expression of 4-repeat tau has been observed, extensive culture (150–365 days) was required [41]. The tau profile observed in neurons generated by transdifferentiation has not, to our knowledge, been reported and while alterations in tau phosphorylation have been observed [4, 38], such modifications of tau have also been demonstrated in iPSC-derived neurons [11, 42]. Whether the ageing profile of neurons derived either from iPSCs or via transdifferentiation protocols is a key limitation in the study of sAD remains to be determined. It is possible that the lack of mature tau isoforms may prevent the activation of key toxicity pathways involved in AD and that the development of more mature neurons is required before accurate modelling of sAD pathogenesis can be achieved.

### The Role of Glial Cells in Modelling sAD

Neurons do not exist in isolation in the brain and there is compelling evidence that glial cells, particularly microglia and astrocytes, are implicated in the onset and progression of AD. Until recently, the ratio of neurons to glial cells in the brain was suggested to be around 10:1 but this ratio may be more like 1:1 and vary between brain regions [43]. While it would be helpful if a consensus could be reached, it is undeniable that glial cells play a role in the onset and progression of AD. Whether the role of glial cells in sAD is distinct from that in fAD has yet to be determined but evidence from the identification of AD risk genes enriched in glial cell types, e.g. *APOE4* in astrocytes and microglia and *TREM2* in microglia, suggests that this may be a key area for investigation. In this section, we will discuss the role of glial cells, focussing on their role in AD, and whether co-culture and/or three-dimensional (3D) culture models may be more physiologically relevant to aid in our understanding of the mechanisms involved in the onset and progression of sAD.

#### Astrocytes

Astrocytes maintain a range of functions but are crucially involved in the clearance of metabolites and toxins, including the clearance of Aβ. Astrocytes, which exist in a quiescent state, can become reactive in AD in response to increasing Aβ. This results in molecular and morphological changes causing alterations in their function including the altered uptake and metabolism of Aβ, changes in calcium homeostasis leading to calcium overload and a reduction in the clearance of glutamate and other neurotransmitters resulting in synaptotoxicity [44]. While the AD field has to date, been somewhat neuron-focussed, the implications of astrocyte dysfunction in AD suggest that addition of astrocytes to neuronal cultures would be of real benefit in our attempts to model sAD. Critically, as discussed above, the mechanism of increased Aβ levels in sAD is likely due to decreased Aβ clearance and metabolism, rather than increased production. As astrocytes express and secrete the majority of known Aβ-degrading enzymes (including NEP and IDE) and are responsible for a large part of extracellular Aβ uptake and breakdown [45], astrocyte impairment in AD may significantly contribute to the development and progression of sAD pathology [46]. Studies on astrocytes have also demonstrated a role in the production of Aβ, potentially through an increase in BACE1 activity in reactive astrocytes indicating that in addition to decreased clearance, astrocytes could also play a role in sAD by increasing Aβ production [47]; although this goes against the hypothesis that sAD is due to decreased clearance of Aβ. Detection of Aβ from single astrocytes demonstrated a sub-population of cells with high levels of secreted Aβ [48], suggesting that sub-populations of astrocytes may play conflicting roles in AD. In support of a role for astrocytes in sAD is their production of the ApoE protein, the E4 isoform of which has a clear genetic link to sAD, which will be discussed in further detail in “Building Better Models for sAD” section.

Several differentiation protocols to produce astrocytes from iPSCs have been published [49–53], including direct conversion to iAstrocytes [54]. While these protocols generate astrocytes with the expected morphology and appropriate expression of astrocytic markers, questions remain around their maturity [51] and their relevance for accurately modelling the changes that occur in sAD. Astrocytes have been generated from iPSCs from patients with fAD with *PSEN1* mutations; *PSEN1* ΔE9 [47], and *PSEN1* M146L [55]. There was no difference in the astrocytic differentiation between the control and *PSEN1* ΔE9 cell lines, but a role for astrocytes in AD pathology in the fAD lines was observed, including increased Aβ42 and calcium dysregulation, as well as changes in metabolism that resulted in increased oxidative stress [47]. Differences in astrocyte, but not neuron, morphology, during differentiation were observed between the control and *PSEN1* M146L cell lines and this study also demonstrated alterations in morphology of astrocytes derived from iPSCs from patients with the *APOE4* allele, a genetically defined sAD model [55]. This has recently been further supported by altered gene transcription in iPSC-derived astrocytes between isogenic APOE3/E3 to E4/E4 cell lines [56]. Astrocyte dysfunction was also observed in astrocytes generated from an *APOE4* cell line compared to a control in their role in Aβ degradation and clearance [57] with impaired Aβ uptake [56].

#### Microglia

Microglia play a distinct role as the innate immune cells of the CNS and in AD have been shown to accumulate as a result of increased Aβ levels, to promote Aβ clearance and prevent the formation of amyloid plaques. However, during disease progression, down-regulation of key genes as a result of proinflammatory cytokine production results in a reduction in Aβ clearance, promoting Aβ accumulation and the formation of amyloid plaques [58, 59]. Genetic risk factors for sAD have identified some key genes enriched in microglia; *TREM2, CR1, CD33 and MS4A* have been identified through GWAS and expression of *APOE4* has also been shown to cause phenotypic changes in microglia including impaired Aβ uptake and inflammatory gene activation [56, 60].

Differentiation protocols for derivation of microglia from iPSCs have been difficult to develop due to the distinct developmental origin of microglia, but recently protocols have emerged to produce cells with transcriptomic profiles highly similar to human adult microglia [61–64]. These iPSC-derived microglia-like cells have been used successfully with cortical neurons in 2D [64] and in 3D organoid co-cultures [62, 65], where the microglia-like cells infiltrated the neuronal organoid [65], responded to the neuronal environment [62] and mediated an inflammatory response [64]. In addition, these iPSC-derived microglia-like cells were able to internalise both fibrillar Aβ and brain-derived tau oligomers [62].

#### Oligodendrocytes

Oligodendrocytes generate the myelin sheaths around axons; however the idea that they are just the myelinating cells of the CNS has been recently revised with the suggestion that a subset of proliferative, immature oligodendrocytes may play a role in CNS functionality including in neural repair [66]. Aβ oligomers caused a decrease in myelin proteins [67] and were toxic to oligodendrocytes [68]. There is also evidence that changes in the morphology of oligodendrocytes is altered in AD [69] suggesting that oligodendrocytes may too have a role to play in the onset and progression of AD. Oligodendrocytes have been successfully generated from iPSCs [70–72] but the evaluation of the role they may play in AD, and their relevance in sAD models has, as yet, not been reported.

#### Cells of the Neurovascular Unit

The role of the NVU (reviewed by Ladecola [73]) in neurodegenerative disease, including in AD, has recently attracted significant attention, and the role of neurovascular alterations in disease has become more widely appreciated. In AD this relatively new direction of research links together clinical observations; the existence of ischaemic lesions with AD pathology, links between vascular dementia and AD, and the role of neurovascular dysfunction in patients with cerebral amyloid angiopathy (CAA), which have begun to demonstrate the importance of the NVU [74]. Endothelial cells [75] and mural cells (vascular smooth muscle cells and pericytes) [76] can be generated from iPSCs. With increasing interest in the role of the NVU, the generation of in vitro cell models to allow effective investigation into the role of the NVU in AD are being developed. By definition of the NVU, these models use co-culture of different cell types, with successful quadruple-culture of endothelial cells with neural stem cells, astrocytes and pericytes reported [77]. Modelling of the NVU has also generated interest from a biomaterials perspective, and three-dimensional (3D) models of the NVU are in development [78].

### Neuron and Glial Co-culture Models

While development of robust protocols to generate neurons and glial cell types continues, recent approaches have started to explore the co-culture of different cell types to better understand the interplay between them. Culture of human neurons with rodent astrocytes to aid growth, support and maturation of the neurons has been utilised successfully [11]. Modification of the extracellular matrix by neurons and astrocytes regulated the formation, maintenance and function of the synapse (reviewed by Dzyubenko et al. [79]) highlighting why maturation of iPSC-neurons may be quicker in the presence of astrocytes. However, while this is of benefit in neuronal model development, the incorporation of glia into neuronal cultures derived from iPSCs from sAD patients may reveal distinct phenotypes and indicate novel research avenues not identified by culturing neurons, or indeed glial cells, in isolation. As discussed above, all of the glial cell types have been shown to potentially play a role in the disease, indeed reactive gliosis, the activation and proliferation of glial cells, as a response to neuronal damage has been seen in all neurodegenerative diseases [80].

Due to their common neuroepithelial origin, spontaneous differentiation of astrocytes in neuronal cultures has been observed with extended culture at around day 70 post neural induction [23, 39]. Therefore neurons generated using these methods may exist in co-culture with astrocytes, dependent on the length of culture. More directed co-culture methods can be applied with the most simplistic models involving the co-seeding of two, or more, cell types. Co-seeding of iPSC-derived neurons and astrocytes has been employed demonstrating improved functional maturation of neurons with astrocyte co-culture in both 2D [81] and in 3D organoids [49]. A co-seeding approach allows cells to be seeded in more relevant ratios, although as discussed previously, glial cell number in the brain is regionally specific and is still not clearly defined. More complex systems are available, including culture on transwell inserts and in microfluidic chambers which allow for more subtle investigation of the interaction of different cell types, particularly with regard to the secretion of vesicles and/or soluble factors and their effect on a secondary cell population. Development of co-culture in 3D adds an additional layer of complexity to potential co-culture models. Current approaches for 3D modelling will be discussed in the next section, but as described above, successful co-culture of iPSC-derived microglia [62, 65] and astrocytes [49] with 3D cortical organoids has been used. The development of cell models which explore either the direct physical interactions or the biochemical interactions between different cell types will no doubt further our understanding, but while generation of these co-culture models may be difficult, characterisation of the role of each cell type in co-culture may prove to be just as challenging.

### Three-Dimensional Neuronal Culture

While there are a number of different neuronal differentiation protocols, the majority involve the ‘two-dimensional’ culture of cells on coated wells of cell culture dishes. It has been proposed that a 3D culture model would better recapitulate the in vivo environment promoting the maturation of neurons, suggesting that 3D culture could potentially resolve one of the greatest challenges in the use of iPSC-derived neurons for studying age-related diseases. In addition, the characterisation of AD relies on the identification of Aβ plaques and NFTs, while neurons are maintained in 2D culture with regular replacement of cell culture media, deposition of Aβ and the formation of NFTs cannot be observed under culture conditions. The advantage of 3D culture may therefore not only be to help to accelerate neuron (and glia) maturity but also allow the accumulation of these pathogenic proteins in vitro.

3D culture, in vitro, can be broadly categorised in to two groups: 3D culture using cells in a scaffold-based gel structure and self-organising scaffold-free structures such as organoids. Neuronal organoid cultures derived from iPSCs through embryoid body formation have gained momentum in recent years with a number of protocols being developed [39, 82–84]. The benefits and limitations of these organoids have been recently well reviewed [8] and studies are now emerging using these organoids to model AD using fAD [26] and sAD cell lines [85, 86]. Of note, the analysis of the 3D organoids from sAD patient lines included proteomic profiling which was directly compared with brain tissue proteomics identifying key proteins in axonal injury and oxidative stress in the AD brains and 3D neuronal cultures compared to controls [86]. Interestingly in terms of potential treatment development, it was also demonstrated that the 5 AD cell lines used displayed a differential response to BACE1 and γ-secretase inhibitors.

Scaffold-based 3D neuronal cultures for the study of AD currently appear to be more limited, but commercially available, scaffold-based gels such as matrigel [87–89] and puramatrix [90] have been used successfully for iPSC-derived neuronal culture. Other hydrogel scaffolds have also been developed, including collagen [91], silk-based scaffolds [92], and synthetic hydrogels [53, 93]. Such hydrogels are easily tuneable and can be supplemented with additional extracellular matrix proteins, providing the potential for investigation of the influence of specific proteins in AD, such as heparan sulphate proteoglycans which have been shown to promote amyloid pathology. Modulating the gel matrix to promote amyloid pathology may be a useful tool in the study of sAD as, to date Aβ deposition has not been shown in genetically unaltered iPSC-derived neurons. Aβ deposition was seen in 3D culture of human neural progenitor cells in matrigel, however, these cells were overexpressing both *APP* and *PSEN1* mutations [87, 88]. Interestingly in these cells culture in 3D was also seen to increase 4-repeat, adult tau compared to 2D culture [87]. Whether culturing of iPSC-derived neurons from sAD patients will enable the development of AD pathology in vitro, remains to be seen. It is possible that both co-culture and 3D culture need to be combined to better recreate the neuronal environment to allow both the physical and biochemical connections needed. More complex structures within hydrogel matrices may be a way to develop such models and the development of technologies such as 3D bioprinting may enable such models to be generated [94].

An alternative to in vitro 3D models has recently been suggested with the proposal of a human-mouse chimeric model to provide a ‘natural 3D culture environment’ and the characterisation of this model for AD [95]. In this work, neural progenitor cells were grafted into the brains of a transgenic AD mouse model, thus exposing them to Aβ deposits and the resultant neuroinflammatory responses produced by the AD mouse. The effect of this culture environment resulted in neuronal loss that was seen only with grafted human neurons and not with grafted mouse neurons. One key finding from this work was the presence of a significant amount of 4-repeat tau at 6–8 months post transplantation indicating that this ‘natural’ 3D environment can evoke accelerated neuronal maturation and subsequent abnormal tau phosphorylation, albeit in the absence of tau tangle pathology [95]. This work provides an alternative strategy for investigating AD using human neurons, but how widely this model will be utilised, given its technical complexity remains to be seen.

### Genetic Stratification for sAD

Identification of a large number of genetic risk factors associated with sAD gives a strong indication of the complexity of the disease. This suggests that initiation of sAD may not always be driven by identical mechanisms, which may explain the inconsistencies observed between cell lines from different sAD patients [11]. We propose that genetic stratification of sAD lines and their association with particular disease phenotypes may progress our understanding of sAD onset, and enable more consistent data to be obtained.

The variability in iPSCs from different donors is known to be due to the genetic variation between individuals, rather than differences in the production of iPSCs, particularly as a result of the cell type or origin due to residual epigenetic signatures [96–98]. Large scale studies from the Next Gen consortium, have demonstrated in over 300 iPSC lines from over 100 individuals that around 50% of the variation is due to genetic differences [98] which is in agreement with an independent study utilising over 700 cell lines from over 300 individuals [99]. This overwhelming evidence of the genetic variation identifies the importance of genetic stratification for the study of disease and highlights the limitations of existing studies using a small number of lines from a select number of individuals without genetically matched controls. While this implies that there is still much work to be done to accurately model complex diseases such as sAD, the use of large study cohorts to overcome these limitations and identify key genes [99, 100], including the validation of GWAS variants [101] and functional genetic variation [102] is extremely promising for the future.

A significant factor in the genetic stratification of cell lines will be the generation of isogenic control lines. Variability exists in control cell lines as well as in sAD cell lines, and it is possible that control cell lines may contain a genetic risk factor for sAD. Such variability creates an amount of ‘noise’ in the system that renders the analysis of data almost impossible with very little likelihood of ever achieving statistical significance. The creation of isogenic control lines will enable a direct comparison to be made and will limit the number of cell lines needed for analysis of any particular risk factor. The discovery of CRIPSR/Cas9 gene editing has made the generation of isogenic cell lines possible. CRISPR/Cas9 gene editing has been used to create isogenic control lines from fAD models with mutations in *APP* [103], *PSEN1* [104, 105], and *PSEN2* [103]. In addition, fAD mutations in *APP* and *PSEN1* have also been introduced into a control cell line [106]. However, the use of this technology with iPSCs is not straightforward [106–108] and the effort to generate these isogenic controls should not be underestimated.

Identification of proteome changes in iPSC-derived neurons from sAD cell lines has been demonstrated, with changes in proteins encoded by genes identified by GWAS, including proteins involved in Aβ production and its clearance [28]. The study highlights the use of proteomics and the analysis of protein interaction networks to observe early molecular changes in sAD that may contribute to disease progression, indicating that such methods may be valuable in the identification of a patient-specific cause of disease [28]. While such studies are encouraging and indicate that genetic stratification for sAD is worth pursuing, the validation of the identified genetic risk factors is still needed to enable the field to focus on those known to impact on disease onset and progression. Highlighted below are some of the key genes identified as risk factors for the development of sAD, discussed in terms of the two mechanisms identified in the “Investigating sAD Using iPSC-Derived Neurons” section: Aβ production or Aβ clearance. We have not aimed to provide an exhaustive list of risk genes associated with sAD, but discuss validated genes and existing studies in iPSC-derived models.

### Genetic Risk Factors Altering Aβ Production

Increased Aβ as a result of alterations in APP processing have been clearly demonstrated, through studies on the fAD-associated mutations in *APP* and *PSEN1*, to lead to AD pathogenesis. Several of the genetic risk variants associated with sAD have also been shown to cause an increase in the production of Aβ. Mutations in *ADAM10*, the enzyme primarily responsible for the α-secretase cleavage of APP, decreased α-secretase cleavage of APP resulting in a concomitant increase in β-secretase cleavage causing increased Aβ production [109]. A number of genes have also been implicated in increased Aβ production via alterations in the localisation and trafficking of both APP and the secretases. *SORL1* variants, identified as a risk gene for sAD by GWAS, altered APP trafficking causing increased Aβ [110], while genetic variants of *PICALM, BIN1* and *CD2AP*, also identified as risk genes for sAD by GWAS, demonstrated altered trafficking of APP due to altered clathrin-mediated endocytosis [111]. *PICALM* also affected the trafficking of γ-secretase resulting in alterations in the Aβ42:40 ratio [112]. Aβ production can also increase as a result of increased APP expression, such as is seen with *APP* duplication in Downs’ syndrome. Both *APOE* [113] and *CLU* [114], genes identified as risk factors for sAD, altered APP expression. *APP* transcription has been shown to be stimulated by *APOE* in an allele dependent manner [113], while decreased levels of clusterin, which like APOE regulates cholesterol metabolism, have been shown to increase APP expression [114]. Variants of *CLU* that decrease its functional capacity would be expected to increase APP expression, thereby increasing Aβ production.

### Genetic Risk Factors Altering Aβ Clearance

Several identified risk genes for sAD mediate their effect by decreasing Aβ clearance. *APOE* and *CLU*, which both play a role in cholesterol uptake, are involved in the uptake and clearance of Aβ [115]. Cholesterol depletion resulted in a decrease in Aβ uptake and clearance. APOE also mediates clearance of Aβ through the BBB which is mediated by the Aβ chaperone proteins, receptor for advanced glycation end products (RAGE) and low-density lipoprotein receptor related protein-1 (LRP1). Mutations in *LRP1* have been identified, and *PICALM*, identified as a risk factor for sAD, has a role in mediating LRP1 function, suggesting an additional role for PICALM [112]. An iPSC-derived endothelial cell model demonstrated that a protective mutation in *PICALM* resulted in increased clearance of Aβ [112]. Aβ clearance can also occur through phagocytosis and mutations in *ABCA7*, a lipid transporter that plays a role in the phagocytic removal of Aβ has also been identified as risk gene for sAD [116]. Decreased proteolytic Aβ degradation has also been implicated, as NEP expression is decreased with age and associated with the accumulation of Aβ plaques [24]. *NEP* polymorphisms increased the risk of sAD [117, 118] and identified polymorphisms in *IDE* also increased the risk of developing sAD [119].

### Using iPSC-Derived Models with Genetic Risk for sAD

*APOE* is arguably the most well-studied gene in sAD with several possible pathological functions including in mitochondrial dysfunction, decreased lipid, cholesterol and glucose metabolism, increased tau phosphorylation, increased neuroinflammation and, as indicated above, increased Aβ via increased APP expression and decreased clearance [18, 19, 120–122]. In iPSC-derived cholinergic neurons from sAD patients with an *APOE4* allele, several phenotypic changes were observed. The APOE3/E4 genotype of the sAD patients resulted in neurons with more susceptibility to neurotoxic stimuli, and increased calcium levels resulting in neuronal excitotoxicity compared to control neurons [123]. APOE4 expression in iPSC-derived neurons has since been shown to result in increased Aβ production and increased tau phosphorylation causing GABAergic neuron degeneration [124]. Linked to APOE, the expression of SORL1, a neuronal APOE receptor, was decreased in iPSC-derived neurons carrying the *APOE4* allele, resulting in increased Aβ [125]. Protective variants in *SORL1*, have also been shown, in iPSC-derived neurons from sAD patients and controls (including cell lines used in Israel et al., Gore et al., and Woodruff et al., to decrease Aβ levels [11, 25, 30, 126]).

Despite being the largest genetic risk factor for sAD, the presence of *APOE4* does not guarantee AD onset, indicating that interaction with additional genetic or environmental risk factors may be involved. Indeed there are many identified risk genes that associate with each other and with specific environmental risk factors. However, the studies in iPSC-derived models, to date, indicate that specific phenotypes exist as a result of genetic variants and highlight that genetic stratification of sAD cell lines may be beneficial both in understanding the disease and for targeting therapeutics. While this is certainly promising, significant progress still needs to be made to validate identified genetic risk factors, stratify iPSC lines, identify the key cell type through which each risk factor may mediates its effects and understand the extent to which susceptibility genes interact with each other and with environmental factors.

## Environmental and Genetic Risk Factors

The lack of a clear genetic cause suggests that the development of sAD is also heavily influenced by environmental risk factors. Here, we define environmental risk factors to mean a non-genetic, external modifier. At present there are several factors that have been identified as increasing the risk of developing sAD. This section looks briefly at what these environmental risk factors are, how they contribute to AD pathology, and whether they have, or can be, modelled using iPSC-derived cell lines to progress our understanding of sAD. We also identify where key risk genes have been identified linking to these specific environmental factors to highlight where a combined approach of genetic stratification and modulation of an environmental risk factor may help us progress our models of sAD.

### Ageing

Ageing is by far the largest risk factor for the development of AD, but the effects are complex, difficult to define and are still not completely understood. Age-related changes result in increased Aβ deposition; for example, failure of perivascular drainage results in deposition of insoluble Aβ in arterial walls that prevents the removal of soluble Aβ, possibly due to the ageing and stiffening of arteries and the build-up of fibrillar Aβ [127]. As discussed previously, the epigenetic changes that occur with ageing are lost in the reprogramming of iPSCs; therefore, aside from using transdifferentiation protocols to maintain such changes, the use of iPSC-derived cell models will require stimulation of ageing through identified age-related proteins (e.g. progerin or inhibition of klotho) or by cell stress to induce premature ageing. Progerin promotes premature ageing and iPSC-derived smooth muscle cells from a patient with Hutchinson-Gilford Progeria syndrome have been shown to have markers of premature ageing following differentiation [128]. Klotho, an anti-ageing protein, protects neurons from oxidative stress [129] and its expression reverses ageing; klotho deficiency may therefore promote ageing. Indeed, when klotho expression was deficient, an accelerated ageing phenotype was observed due to activation of wnt signalling resulting in the depletion and senescence of stem cells [130]. Klotho has also been shown, in mouse models, to mediate autophagy which may have additional implications on Aβ deposition. While modulation of progerin and klotho may provide a way to age iPSC-derived models for the study of sAD, cell stress enables the stimulation of ageing without requiring genetic modification.

### Oxidative Stress

Oxidative stress, or the generation of ROS, results in a multitude of damaging cellular effects. While the overall effect on cellular ageing is still to be investigated, mitochondria are considered to be the largest contributors of ROS [131] and both oxidative stress and mitochondrial dysfunction (discussed below) are strongly implicated AD as well as other neurodegenerative diseases [132]. Oxidative stress plays a role in Aβ degradation and clearance (reviewed by Cheignon et al. [133]). As previously described, iPSC-derived astrocytes from fAD patients showed increased levels of oxidative stress [47] and iPSC-derived neurons from both fAD [47] and sAD [3] patients were more reactive to H_2_O_2_ treatment. There are several genetic risk genes that may affect the neuronal response to oxidative stress including *BMI1*, expression of which decreased in the AD brain, and more recently in iPCS-derived cortical neurons from sAD patients [134]. Given the links of oxidative stress to other genetic and other environmental factors (as described below), induction of oxidative stress may offer a solution to induce AD in an iPSC-derived sAD model.

### Hypoxia

Hypoxia has been proposed as a mechanism contributing to AD pathology and conditions causing hypoxia, such as stroke, ischaemic injury, cardiac arrest and neurovascular disease, are identified risk factors for the development of AD [135, 136]. Hypoxia caused increased Aβ deposition in AD mouse models [137–139] and this effect was shown, both in cells and in AD mouse models, to be regulated by an increase in γ-secretase activity resulting from the direct interaction of γ-secretase with hypoxic-inducible factor 1-α (HIF-1α) [140], or via the demethylation of the gene encoding γ-secretase [141]. Activation of HIF-1α by mitochondrial ROS production upregulated BACE1 expression, increased BACE1 activity and thereby contributed to increased Aβ deposition in hypoxia [142]. Clearance of Aβ may also be impaired as the Aβ-degrading enzymes endothelial converting enzyme 1 (ECE-1) and NEP were downregulated during hypoxia, while conversely IDE was upregulated by hypoxia [143–146]. Hypoxia also resulted in increased tau phosphorylation at several sites in the hippocampus [90, 147] but whether this occurs as a direct effect of increased Aβ levels has not been confirmed.

The application of hypoxic conditions in iPSC-derived sAD models has not been widely explored. Hypoxia has been widely utilised for the generation and maintenance of pluripotency, and also for the directed differentiation of iPSCs to a neural fate [148–150]. Using hypoxic conditions during the differentiation stage of iPSC-derived cells may affect maturation of different cell types which could be of benefit when deriving iPSC neurons or astrocytes, where the maturity of these cells is of concern. Post differentiation, the use of hypoxia to simulate an environmental risk factor with well characterised AD pathology may be an excellent method for cell modelling due to the relative ease with which the severity of oxygen deprivation can be modified to mimic either acute injury (i.e. stroke) or chronic conditions (i.e. ageing) by adjusting both oxygen conditions and length of exposure.

### Inflammation

Neuroinflammation clearly plays a role in AD and other neurodegenerative diseases, although whether it is harmful or protective is still a source of debate. It is possible, however, that it plays both roles and that the effect of inflammation may be cell dependent. Microglia and astrocytes both mediate an immune response in the brain. Microglia can be activated to become anti-inflammatory, where debris is cleared through phagocytosis, or pro-inflammatory, with the release of ROS and cytokines [151]. Astrocytes which become reactive during cell stress are traditionally considered to have a neuroprotective role. However, in conditions mimicking neuroinflammation, astrocytes lose many of their normal functions and become destructive [152]. Neuroinflammation was originally considered a secondary effect of AD due to the build-up of Aβ plaques and NFTs, but increasing evidence suggests that inflammation contributes to the early development of disease [153]. Pertinent to a key role in sAD, several risk genes associated with the inflammatory response have been identified, including *TREM2* which has been investigated in iPSC-derived neurons, albeit in a model of frontotemporal dementia [65]. Induction of a pro-inflammatory environment to mimic changes occurring in AD can be easily achieved using cytokines, and the use of such models have been well described, although not, to date, in iPSC-derived cells. iPSC-derived neurons, however, have been treated with H_2_O_2_ and NO to model oxidative stress and inflammation, where viability was dramatically reduced. This was further exacerbated in both sAD and fAD lines. Notably, apigenin which may inhibit NF-κB activity, protected against inflammation induced cell death [154]. For future models of inflammatory responses in iPSCs the influence of both astrocytes and microglia need to also be assessed, especially as these cells play such an important role in Aβ degradation and clearance.

### Metabolic Changes

Changes in energy metabolism are a key characteristic observed in ageing and in the early stages of AD. Longitudinal studies have also demonstrated links between AD and metabolic disease, including Type 2 diabetes mellitus (T2DM) and metabolic syndrome (MetS) [107], and in particular have demonstrated that obesity is a clear risk factor for AD. Obesity causes a low-grade inflammatory response, due to activation of PPR, IKKβ and NF-κB, which results in metabolic dysfunction (reviewed by Lumeng and Saltiel [155]). In addition, obesity also results in changes in glucose availability and insulin signalling [156], which are also altered in both T2DM and MetS. Changes in both glucose and insulin can cause alterations in Aβ levels which may lead to the development of sAD. The Aβ-degrading enzyme IDE preferentially binds insulin over Aβ and when insulin levels are high, as happens in the early stages of T2DM and MetS, the increased insulin results in competitive inhibition of Aβ binding and its degradation by IDE [157, 158]. Conversely, it has been demonstrated in a mouse model which does not produce insulin, that when insulin levels were low, such as occurs in later stage diabetes, IDE expression was significantly decreased [159]. IDE can also be influenced by alterations in glucose levels, first as changes in glucose levels result in associated changes in insulin levels, and second, because high glucose levels can directly inhibit IDE activity through s-nitrosylation [157]. The dysregulation of insulin and glucose in T2DM and MetS demonstrates how a potential environmental risk factor may cause sAD; whether such changes can be utilised to drive the onset of sAD in iPSC-derived models, remains to be seen.

### Mitochondrial Dysfunction

Part of the metabolic changes associated with an increased risk of sAD are changes in oxygen consumption which link to mitochondrial function. Mitochondria play a vital role in cellular energy production and are also involved in apoptosis and calcium homeostasis. Mitochondrial dysfunction resulting from alterations in mitochondrial membrane proteins, have been linked with AD due to alterations in APP expression and APP processing which lead to increased Aβ accumulation. Furthermore, mitochondrial dysfunction leads to increased oxidative stress and inflammation and has been shown to increase tau phosphorylation, although whether these effects directly cause sAD or are an associated change as a result of disease onset has not been fully elucidated (reviewed by Swerdlow et al. [160]).

Mitochondrial dysfunction has been explored in several different iPSC-derived neuronal models of neurodegeneration. In iPSC-derived neurons from sAD patients, mitochondrial dysfunction was observed as a result of increased formation of oxidative phosphorylation chain complexes resulting in increased ROS production [29]. In addition, it has been hypothesised that the increased sensitivity to ROS that has been observed in iPSC-derived neurons from sAD patients is due to mitochondrial stress [12]. Mitochondrial dysfunction, leading to oxidative stress, was observed in iPSC-derived neurons from patients with familial mutations in *PINK1, PARK2* and *TAU* that cause either Parkinson’s disease or frontotemporal dementia [161–163]. This interestingly links to studies from iPSC-derived neurons from a fAD patient with a *PSEN1* mutation where decreased mitophagy and autophagy have been reported due to changes in PINK1 and PARK2 [164]. These studies indicate that mitochondrial dysfunction could initiate the onset of sAD by triggering a number of key pathways and responses in iPSC-derived cell models.

## Future Directions and Outstanding Questions

**Fig. 1:**
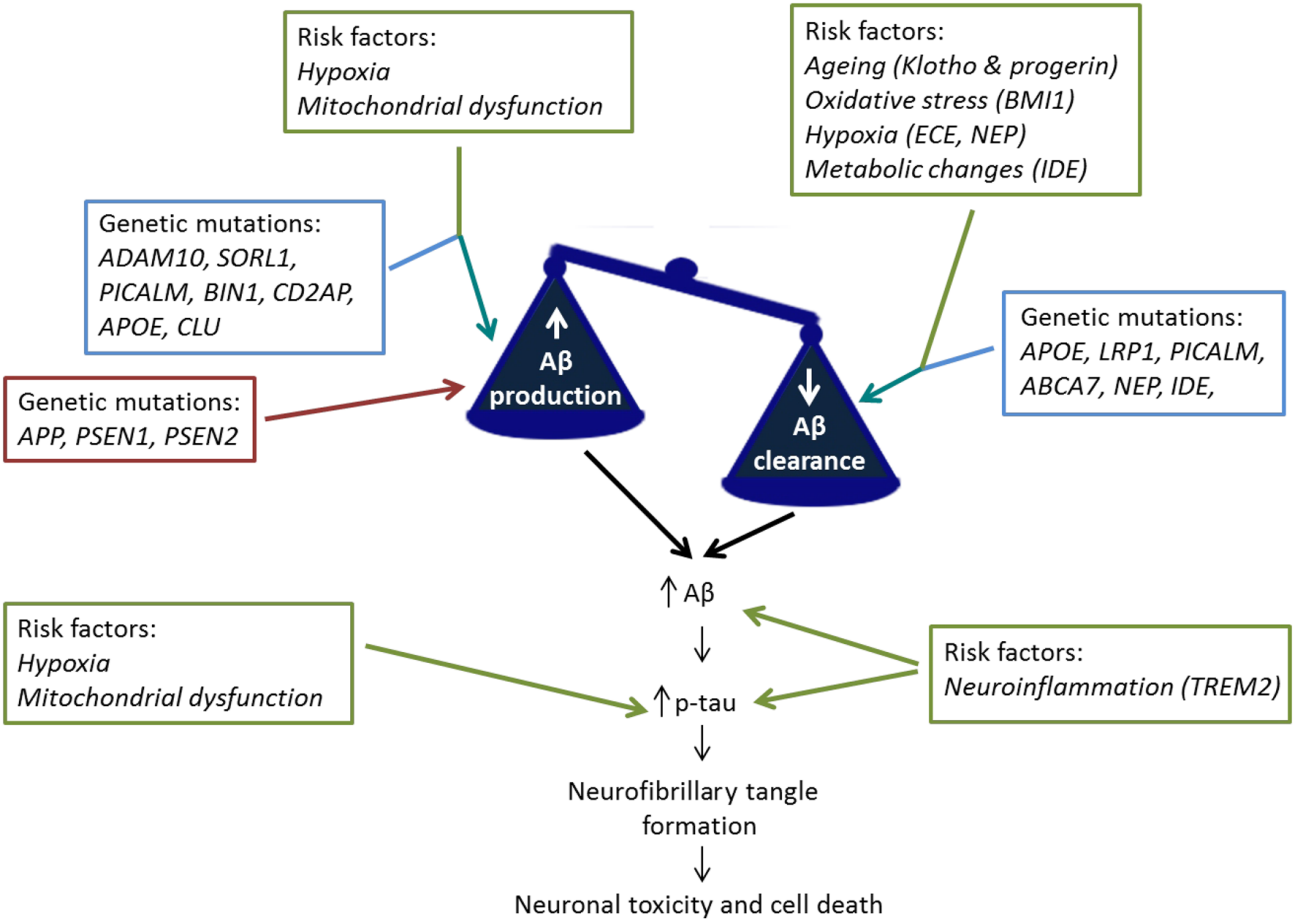
Modelling sporadic Alzheimer’s disease. fAD  is caused by genetic mutations that drive increased Aβ production. In contrast sAD is caused by multiple linked factors that disrupt the balance between Aβ production and Aβ clearance. These factors include genetic mutations  and environmental and genetic risk factors . We propose that more successful models of sAD using iPSC-derived cell lines may be generated using genetic stratification of patient lines, along with the use of environmental and genetic risk factors to initiate disease onset

Research using defined iPSC-derived neuronal models from fAD patients demonstrates the success of this approach to investigate AD. The measurable changes that can be observed in Aβ, tau and related proteins in neurons derived from fAD cell lines have enabled further understanding of the onset and development of pathology in human neurons which is of great benefit to the field. In contrast, however, despite encouraging elements in the analysis of AD pathology in iPSC-derived neurons from sAD patients, the inconsistency in the production of an ‘AD phenotype’ suggests that further refinement of these models is required to allow us to fully investigate the causes and mechanisms of the disease and its progression. We have summarised the factors described in this review that need to be considered in modelling sAD in Fig. 1.

The future of using iPSC-derived models in the study of sAD requires several key areas to be addressed, summarised below as four key questions:-

1. Can we recapitulate disease progression by 3D culture of neurons and glial cells?
2. Will genetic stratification of iPSCs by sAD risk genes develop more consistent models?
3. Is Aβ clearance impaired in iPSC-derived sAD models?
4. Does the application of environmental risk factors promote the onset of a sAD phenotype in iPSC-derived neurons?

iPSC-derived models are advancing, including the development of co-culture to include glial cells and 3D culture with the aim to both mature neurons more rapidly and to create a more physiologically relevant model to study the disease. While the use of fAD cell lines in more complex culture models will hopefully start to delineate disease mechanisms, the generation of iPSC lines from sAD patients may still not give consistent results. This lack of consistency has implications not only in developing our understanding of the onset and progression of sAD, but also in the development of treatments, particularly with the move towards personalised medicine. Therefore, we propose that genetic stratification of cell lines may address some of the inconsistencies observed between different sAD iPSC models and such stratification may identify key pathways for investigation in a given cell line. This will, however, require not only the stratification of existing lines, but the development of isogenic controls. In this review, we have discussed genetic stratification in terms of Aβ production vs Aβ clearance to highlight the potential role of Aβ clearance in the onset and progression of sAD. While not neglecting alterations resulting in increased Aβ production, we consider that Aβ clearance in iPSC-derived cell lines from sAD patients is a key area for investigation to determine whether it may be a primary mechanism for the disease. Finally, despite better culture models and genetic stratification of sAD cell lines, it may be that the initiation of the disease requires additional environmental risk factors. The extent to which susceptibility genes interact with each other and with environmental factors may provide further clues to aetiology. Therefore, we propose that the combined use of specific environmental risk factors in cell lines susceptible to such specific changes, based on genetic stratification, may be able to drive sAD onset.
